# Discovery of Avian TCRVβ2^+^ T Cells Recognizing the Endogenous Ganglioside via a chCD1.1‐Dependent Pathway

**DOI:** 10.1002/eji.70302

**Published:** 2026-10-14

**Authors:** Nitin Kamble, Faisal R Anjum, Shahriar Behboudi

**Affiliations:** ^1^ The Pirbright Institute Pirbright Woking UK; ^2^ Bristol Veterinary School University of Bristol Langford UK

**Keywords:** CD1.1, gangliosides, NKT cells, T cells, TCR

## Abstract

This study presents the discovery and first functional characterization of chicken lipid‐reactive Natural Killer T (NKT)‐like cells, resolving a long‐standing knowledge gap in avian immunology. Using purified TCRVβ2^+^ lymphocytes, we demonstrate through rigorous experiments combining shRNA‐mediated gene silencing, pharmacological pathway blockade, primary MACS‐purified co‐cultures, and intracellular cytokine flow cytometry that these cells are activated by CpG‐ODN‐stimulated antigen‐presenting cells (APCs). Activation selectively depends on APC‐driven glycosphingolipid (GSL) biosynthesis, with CpG‐ODN significantly upregulating key biosynthetic enzymes (*ST3GAL1* and *ST3GAL5*) in APCs. Silencing of enzymes involved in this pathway or chemical inhibition of GSL synthesis abrogated T cell activation. We identify GM3 as a potent, functional lipid ligand activating TCRVβ2^+^ lymphocytes. In parallel, IL‐12 and IL‐18 trigger IFN‐γ production from TCRVβ2^+^ lymphocytes, crucially requiring live APC interactions. Upon activation of splenocytes with GM3, the cells expressed *chIL‐2*, *chIL‐4*, *chIL‐17*, *Granzyme A*, and *Perforin*. By establishing functional, nonpeptide lipid‐reactive T cells in chickens, these findings redefine the conceptual framework of avian innate‐like T cell immunology. This discovery unlocks a highly translatable, universal target for lipid‐adjuvanted poultry vaccines to enhance global animal health and food security.

## Graphical abstract

We present the first report of chicken lipid‐reactive, NKT‐like TCRVβ2+ T cells that recognize the endogenous ganglioside GM3 presented by chCD1.1 on live antigen‐presenting cells. This activation induces a rapid cytokine response from avian lipid‐reactive T cells, offering a universal target for lipid‐adjuvanted poultry vaccines.

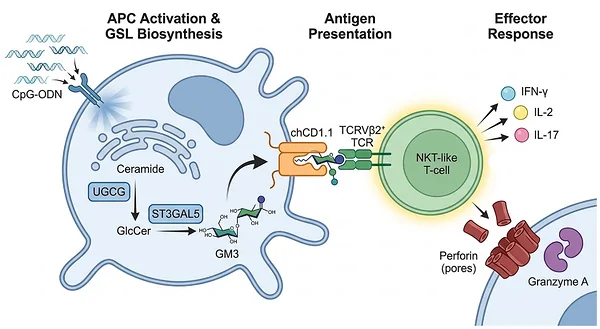

**Abbreviations**
NKT cells: natural killer T cells
GSL: glycosphingolipid
GM3: monosialodihexosylganglioside‐3
CpG‐ODN: CpG oligodeoxynucleotide
PMA: phorbol‐12‐myristate‐13‐acetate
shRNA: short hairpin RNA
APC: antigen‐presenting cells
BMDC: bone marrow‐derived dendritic cells
BMM: bone marrow‐derived macrophages
IL‐12: interleukin 12
IFN‐γ: interferon gamma
ELISPOT: enzyme‐linked ImmunoSpot
MACS: magnetic activated cell sorting
PPMP: DL‐threo‐1‐phenyl‐2‐palmitoylamino‐3‐morpholino‐1‐propanol
NB‐DGJ: N‐(n‐butyl)deoxygalactonojirimycin

## 1 Introduction

Natural killer T (NKT) cells are innate‐like T lymphocytes that recognize lipid and glycolipid antigens presented by CD1 molecules. In mammals, NKT cells play key roles in bridging innate and adaptive immunity, contributing to immune responses against tumors and a wide range of pathogens. These cells typically express a semi‐invariant T cell receptor (TCR), characterized by a restricted TCR α‐chain repertoire paired with limited β‐chain variants, and are therefore often referred to as CD1d‐restricted T cells. Upon activation, NKT cells rapidly secrete diverse immunomodulatory cytokines, including interferon‐γ (IFN‐γ), interleukins (IL‐4, IL‐13, IL‐17A), and other mediators that influence downstream immune responses. Through cytokine secretion and CD40L expression, NKT cells promote dendritic cell maturation and enhance antigen‐specific T and B cell responses [1, 2, 3, 4, 5]. These properties have led to their exploitation in vaccine development, where NKT cell ligands are used as adjuvants to improve immunogenicity [6]. Despite extensive characterization in mammals, the presence and function of NKT cells in chickens remain unclear.

Although structural homologs of mammalian CD1d (CD1.1 and CD1.2) have been identified in chickens and shown to possess lipid‐binding grooves, functional evidence of CD1‐restricted, lipid‐reactive T cells is lacking [7]. Given the importance of vaccination in controlling avian infectious diseases, identification and characterization of NKT‐like cells in chickens could inform the development of improved vaccine strategies. Chicken T cells are classified into two major subsets based on TCRαβ expression: TCR2 (TCRVβ1^+^) and TCR3 (TCRVβ2^+^) [8, 9, 10, 11]. Research has suggested that TCRVβ1+ and TCRVβ2+ T cell lines can share the same Vα gene families, indicating that the distinction between these two lineages is primarily defined by their beta chain usage [10]. While their phenotypic features and tissue distribution have been described, their functional specialization, particularly in relation to innate‐like or NKT‐like activity, remains poorly understood. Here, we report the discovery of a distinct subpopulation of avian TCRVβ2 T cells that recognize endogenous lipid ligands in a chCD1.1‐dependent manner. We demonstrated that only live APCs activate these T cells to produce IFN‐γ. This activation is associated with upregulation of GSL biosynthesis in APCs, and inhibition of enzymes involved in GSL biosynthesis significantly impaired this response. We identified the endogenous or exogenous ganglioside GM3 presented by live APCs as a potent, functional lipid capable of activating TCRVβ2 T cells. Collectively, by establishing a functional, nonpeptide lipid‐reactive T cell response in chickens, these findings refine our understanding of avian mucosal immunology and unlock a novel classical MHC‐independent target for universal poultry vaccines.

## 2 Materials and Methods

### 2.1 Animals and Ethical Approval

Spleens and femurs were collected from 3‐week‐old specific pathogen‐free (SPF) mixed‐sex outbred Rhode Island Red chickens. Birds were euthanized by cervical dislocation, and tissues were aseptically collected in phosphate‐buffered saline (PBS) containing penicillin/streptomycin. The killing of birds was performed according to UK Home Office regulations and approved by the ethical committee of The Pirbright Institute.

### 2.2 Isolation and Stimulation of Splenocytes

Splenocytes were prepared by mechanical dissociation through 40 µm cell strainers (BD Biosciences, Oxford, UK) and isolated by density gradient centrifugation using Histopaque 1.083 (500×*g*, 30 min, 4°C) (Sigma‐Aldrich, Dorset, UK). Cells were washed, resuspended in RPMI‐1640 supplemented with 5% heat‐inactivated fetal calf serum and penicillin/streptomycin (Thermo Fisher Scientific, Loughborough, UK), and adjusted to 5 × 10^6^ cells/mL. Cells were stimulated with PMA (50 ng/mL) and ionomycin (100 ng/mL), CpG‐ODN (1 µg/mL) (InvivoGen, Toulouse, France), or gangliosides GM3 or GM1 (10–50 µM) (Sigma‐Aldrich) at 41°C with 5% CO_2_ for 6 h (gene expression) or 24 h (cytokine analysis).

### 2.3 Isolation of Chicken T Cells

Chicken CD3^+^, TCRVβ1^+^, and TCRVβ2^+^ T cells were isolated from splenocytes using anti‐PE MicroBead kits (Miltenyi Biotec, Woking, UK) according to the manufacturer's instructions. Briefly, splenocytes (1 × 10^7^) were incubated with PE‐conjugated mouse anti‐chicken CD3, TCRVβ1, or TCRVβ2 antibodies (Cambridge Bioscience, Cambridge, UK) for 30 min at 4°C, washed, and subsequently labelled with anti‐PE MicroBeads for 30 min at 4°C. After washing, cells were separated using MACS columns. Purity of the isolated populations (>97%) was confirmed by flow cytometry.

### 2.4 Generation of Antigen‐Presenting Cells

Bone marrow cells were isolated from femurs, filtered through 40 µm cell strainers, and enriched by density gradient centrifugation using Histopaque 1.119. Cells were cultured in RPMI‐1640 supplemented with 5% chicken serum (Sigma‐Aldrich), 2‐mercaptoethanol, and penicillin–streptomycin (Thermo Fisher Scientific). For differentiation, cells were cultured with chicken GM‐CSF (10 ng/mL) and IL‐4 (20 ng/mL) (Cambridge Bioscience) to generate bone marrow‐derived dendritic cells (BMDCs), or with 2% chicken M‐CSF (Pirbright Institute, Surrey, UK) to generate bone marrow‐derived macrophages (BMMs). Cell culture media and cytokines were refreshed on days 1, 3, 5, and 7. Cells were harvested using TrypLE Select (Gibco, Paisley, UK) and used for downstream assays.

### 2.5 Co‐Culture Assays

BMDCs were pretreated with CpG‐ODN, GM3, GM1, or vehicle for 16 h, washed, and co‐cultured with purified T‐cell subsets at a 1:5 ratio in all experiments for 24 h. For inhibition studies, BMDCs were pretreated with nontoxic concentrations of PPMP (Abcam, Cambridge, UK), NB‐DGJ (Cambridge Bioscience) (10 µM, 2 h) or vehicle before CpG‐ODN stimulation, and the inhibitors were maintained during CpG‐ODN stimulation for an additional 16 h. Cells were then washed, and viability was determined using trypan blue exclusion. IFN‐γ production was measured by ELISpot or ELISA. In some experiments, splenocytes or purified TCRVβ1 or TCRVβ2 were stimulated with recombinant chIL‐12, chIL‐18, chIFN‐α, or chIFN‐β for 24 h. For APC co‐cultures, T cell subsets were co‐cultured with BMDCs (1:5) in the presence of chIL‐12 and chIL‐18. BMDCs were fixed with paraformaldehyde before the addition of cytokines and T cells to assess the requirement for APC viability. IFN‐γ production was measured by ELISpot assay.

### 2.6 IFN‐γ ELISpot, ELISA, and Intracellular Assays

IFN‐γ production was quantified using ELISpot and ELISA assays with anti‐chicken IFN‐γ capture and detection antibodies (Thermo Fisher Scientific). For ELISpot, spots were developed on PVDF plates (Merck Millipore, Watford, UK), counted using an automated ELISpot reader (Advanced Imaging Devices GmbH, Germany), and expressed as spot‐forming units per million cells. For ELISA, cytokine concentrations were determined using a recombinant chicken IFN‐γ standard curve. The anti‐chicken IFN‐γ capture antibody was conjugated using an APC conjugation kit (Abcam, Cambridge, UK). Splenocytes were stimulated with GM3, diluent control or phorbol 12‐myristate 13‐acetate (PMA; 50 ng/ml) plus ionomycin (Ion; 100 ng/ml) for 4hrs in the presence of Brefeldin A, and consequently, cells were first stained with the LIVE/DEAD Fixable Aqua Dead Cell Stain Kit (Thermo Fisher Scientific), followed by surface staining with anti‐chicken TCRVβ1‐PE or TCRVβ2‐PE antibodies, and gating for these cells was determined using PE‐conjugated IgG1 isotype control (Cambridge Bioscience). The cells were then fixed and permeabilized for intracellular staining with the APC‐conjugated chicken IFN‐γ. Acquisition was performed on a BD LSRFortessa flow cytometer (BD Bioscience), and the data were analyzed using FlowJo v10.07.

### 2.7 qRT‐PCR Analysis and Gene Silencing

Total RNA was extracted using TRIzol reagent, treated with DNase I, and reverse transcribed into cDNA using a first‐strand cDNA synthesis kit (Thermo Fisher Scientific). Gene expression was quantified using SYBR Green‐based qPCR with Luna Universal qPCR Master Mix (New England Biolabs Ltd, Hitchin, UK) on a QuantStudio 5 system (Applied Biosystems, Paisley, UK). Relative gene expression was calculated as 2^−ΔC^ *^t^*, where ΔC*t* = C*t*_target_ − C*t*_GAPDH_. This inverse exponential relationship ensures that higher bars on the graphs directly correspond to greater mRNA abundance, providing an intuitive visual representation of gene silencing. All primers (Table 1) were validated for specificity and efficacy with a single melting curve peak, expected amplicon sizes, and 95%–105% amplification efficiencies. No‐template controls were included in each run to rule out contamination.

**TABLE 1:** Primers and shRNA sequences.

Name	Sequence (5’–3’ end)
CD1.1 Fwd	ACCTCGATCACAGCTGAGACTGAAGTTCAAGAGACTTCAGTCTCAGCTGTGATCTT
CD1.1 Rev	CAAAAAGATCACAGCTGAGACTGAAGTCTCTTGAACTTCAGTCTCAGCCCGACCTT
CD1.1 scr Fwd	ACCTCGTTGCAGAACGTGACAGATCATCAAGAGTGATCTGTCACGTTCTGCAACTT
CD1.1 scr Rev	CAAAAAGTTGCAGAACGTGACAGATCACTCTTGATGATCTGTCACGTTCTGCAACG
UGCG Fwd	ACCTCGCACTTCATGTCCATCATCTATCAAGAGTAGATGATGGACATGAAGTGCTT
UGCG Rev	CAAAAAGCACTTCATGTCCATCATCTACTCTTGATAGATGATGGACATGAAGTGCG
UGCG scr Fwd	ACCTCGCACTACAGTCTCTCACTATTTCAAGAGAATAGTGAGAGACTGTAGTGCT
UGCG scr Rev	CAAAAAGCACTACAGTCTCTCACTATTCTCTTGAAATAGTGAGAGACTGTAGTGCG
ST3GAL5 Fwd	ACCTCGAGAAATACAGCATGGACATATCAAGAGTATGTCCATGCTGTATTTCTCTT
ST3GAL5 Rev	CAAAAAGAGAAATACAGCATGGACATACTCTTGATATGTCCATGCTGTATTTCTCG
ST3GAL5 scr Fwd	ACCTCGCAAATGATACGAGAGCTAAATCAAGAGTTTAGCTCTCGTATCATTTGCTT
ST3GAL5 scr Rev	CAAAAAGCAAATGATACGAGAGCTAAACTCTTGATTTAGCTCTCGTATCATTTGCG
CD1.1 Fwd	TGCATCTTCCACTGTTGGCA
CD1.1 Rev	TCCGGAAATCCGTTCCAGAC
CD1.2 Fwd	GCCATGGGTCCAAGAGATCA
CD1.2 Rev	CGGCAAGGAGCAGTATGGTG
ST8SIA1 Fwd	GCTTTGGTCTCGAAAAGCAT
ST8SIA1 Rev	CAGGACTTTTCCTAGTCAGTG
ST8SIA5 Fwd	CTCCTAATTGAGCCAGACGC
ST8SIA5 Rev	AATCACTCTGTCCGTGGATGT
ST3GAL1 Fwd	GGCAACCTTCGTCAGTCTCA
ST3GAL1 Rev	GTGCACGGTTCATTCTGAGC
ST3GAL5 Fwd	GTTTGTGATTGGAGGGTGCTT
ST3GAL5 Rev	ATTGTTGTGCTCTCTTTACGC
ChPRF1 Fwd	GCGCAGAATTGGAAAGTGGG
ChPRF1 Rev	CTGAGATCCAGTAGTGCACGC
ChGZMA Fwd	ATTGCAATCTGAAGGGAGGCA
ChGZMA Rev	TCGGGACAGTAGTCTGGGTAG
ChIL17A Fwd	GATGCTGGATGCCTAACCCA
ChIL17A Rev	TGTGGTCCTCATCGATCCTGT
ChIL2 Fwd	TGCAGTGTTACCTGGGAGAAG
ChIL2 Rev	CGGTGTGATTTAGACCCGTAAGA
ChIL4 Fwd	AACTGAGCTCTTATGCAAAGCC
ChIL4 Rev	ATGGTGCCTTGAGGGAGGTG
β‐actin Fwd	TGCTGTGTTCCCATCTATCG
β‐actin Rev	TTGGTGACAATACCGTGTTCA

shRNAs targeting selected genes were designed using siRNA Wizard software, cloned into psiRNA‐EGFP‐Zeo vectors (InvivoGen), and transfected into splenocytes 24 h before CpG‐ODN stimulation by electroporation using a NEPA21 electroporator (Nepa Gene Co., Ltd., Chiba, Japan). Knockdown efficiency was confirmed by qRT‐PCR 6 h after CpG‐ODN stimulation. Cell viability was assessed using trypan blue exclusion and ranged from 78% to 80% across all experimental groups, with no difference in viability between the shRNA and scrambled RNA groups.

### 2.8 Statistical Analysis

Data are presented as mean ± SD. Homogeneity of variance was evaluated using the Brown–Forsythe test and was not significantly violated. Statistical significance was determined using one‐way ANOVA with Bonferroni correction (GraphPad Prism). A *p*‐value < 0.05 was considered significant.

## 3 Results

### 3.1 CpG‐ODN‐Treated BMDCs or BMMs Activate IFN‐γ Production by Chicken CD3^+^TCRVβ2^+^ T Cells

Unmethylated CpG oligodeoxynucleotides (CpG‐ODN) activate mammalian antigen‐presenting cells (APCs) to stimulate innate‐like T cells, including NKT cells, via CD1‐dependent and independent pathways [12]. To assess whether CpG‐ODN activates chicken NKT‐like responses, splenocytes were stimulated for 24 h and analyzed by IFN‐γ ELISpot, revealing increased IFN‐γ production (Figure 1A), although the responding cell population was not identified. Purified CD3^+^, TCRVβ1^+^, or TCRVβ2^+^ T cells did not produce IFN‐γ when directly stimulated with CpG‐ODN (Figure 1B), indicating that APCs are required. Accordingly, bone marrow‐derived macrophages (BMMs) or dendritic cells (BMDCs) were pretreated with CpG‐ODN for 16 h, washed, and co‐cultured with T cells. Vehicle‐treated APCs did not induce IFN‐γ, whereas CpG‐ODN‐stimulated BMMs and BMDCs induced robust IFN‐γ production, significantly enhanced in the presence of CD3^+^ T cells (Figure 1C). Further analysis showed that CpG‐ODN‐activated APCs selectively induced IFN‐γ production in TCRVβ2^+^, but not TCRVβ1^+^, T cells (Figure 1D,E), while neither subset responded to vehicle‐treated APCs. Because BMMs and BMDCs produced comparable effects, BMDCs were used for subsequent experiments.

**FIGURE 1:**
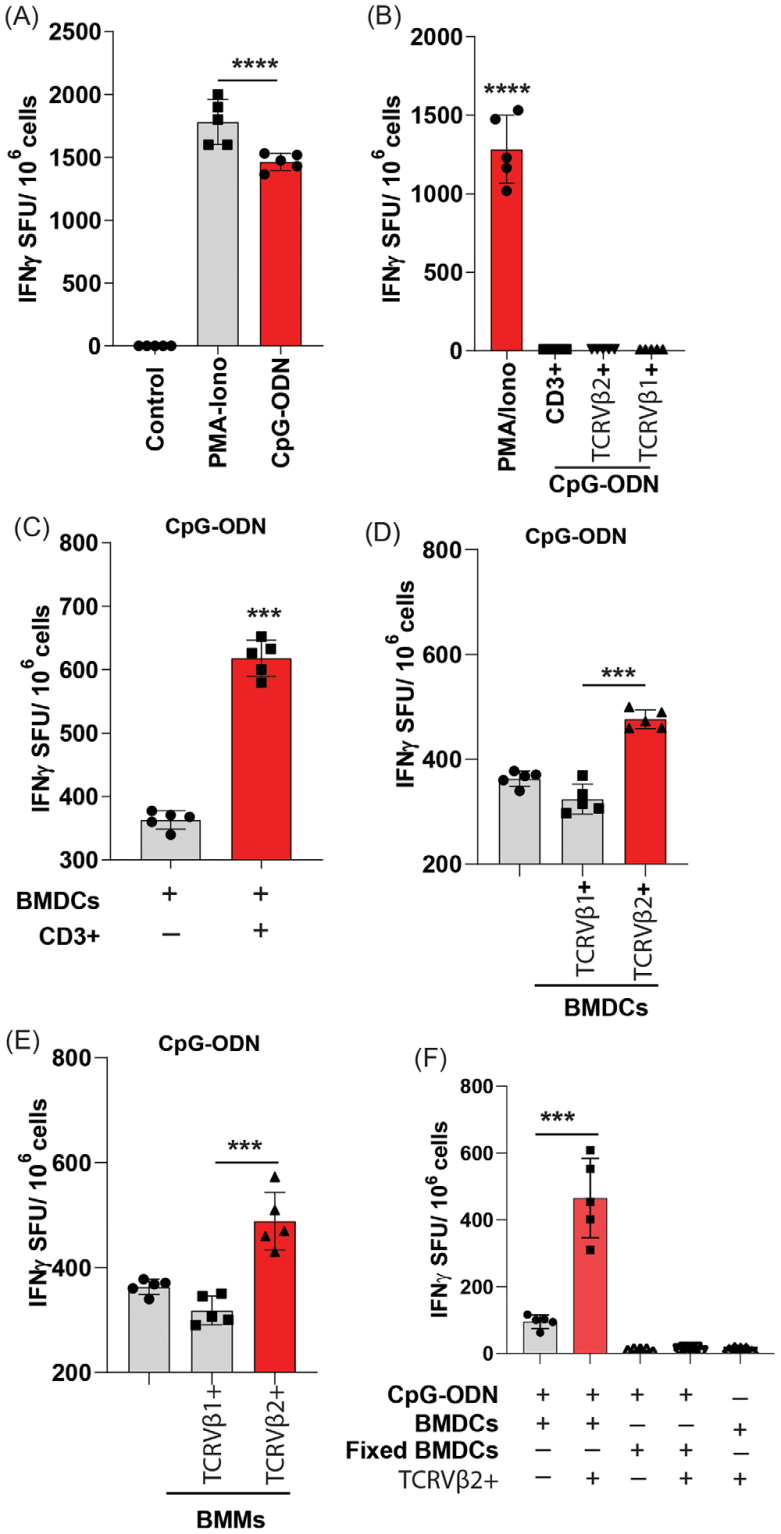
CpG‐ODN‐activated BMDCs and BMMs induce IFN‐γ production in chicken TCRVβ2+ T cells. (A) CpG‐ODN (1 µg/mL) stimulates robust IFN‐γ production in chicken splenocytes. PMA (50 ng/mL) plus Ionomycin (1 µg/mL) served as a positive control. (B) Purified CD3+, TCRVβ1, and TCRVβ2 in response to CpG‐ODN in the absence of APCs. (C) CpG‐ODN‐treated (24 h) BMDCs significantly stimulate IFN‐γ production when co‐cultured with purified CD3+ T cells (1:5 ratio). (D, E) CpG‐ODN‐activated BMDCs (D) and BMMs (E) specifically stimulate TCRVβ2 T cells, indicating a requirement for live APC metabolic activity or processing. (F) CpG‐ODN‐stimulated BMDCs were either fixed or left unfixed after stimulation and then co‐culture with or without TCRVβ2 cells. IFN‐γ production was analyzed using an ELISPOT assay. Data are representative of three independent experiments. Each data point represents an individual chicken (n = 5); bars represent the mean ± SD. Asterisks denote significant differences (***p < 0.001 and ****p < 0.0001) determined by one‐way ANOVA.

To determine whether cell viability was required for activation, CpG‐ODN‐treated BMDCs were fixed prior to co‐culture. Fixed APCs failed to induce IFN‐γ production in TCRVβ2^+^ T cells (Figure 1F), indicating that live APCs are essential for activation. Together, these results demonstrate that CpG‐ODN‐activated APCs specifically induce IFN‐γ production in chicken TCRVβ2+ T cells.

### 3.2 Activation of CD3^+^TCRVβ2^+^ T Cells Is Linked to Increased GSL Biosynthesis in CpG‐ODN‐Treated APCs

To investigate the role of the glycosphingolipid (GSL) pathway (Figure 2A) in CpG‐ODN‐mediated activation of TCRVβ2^+^ T cells, we first analyzed expression of key biosynthetic enzymes in BMDCs. CpG‐ODN stimulation upregulated GM3 synthase (St3gal5) and ST3 β‐galactoside α‐2,3‐sialyltransferase 1 (St3gal1) compared with controls (Figure 2B), indicating activation of the GSL pathway. To assess functional relevance, BMDCs were pretreated with nontoxic concentrations of glucosylceramide synthase inhibitors NB‐DGJ or PPMP before CpG‐ODN stimulation and co‐culture with TCRVβ2^+^ T cells. Both inhibitors significantly reduced IFN‐γ‐producing cells by ∼60% and ∼50%, respectively (Figure 2C). To confirm these findings, UGCG and St3gal5 were silenced using shRNA in splenocytes prior to CpG‐ODN stimulation. Knockdown efficiency was confirmed by reduced mRNA expression (Figure 2D,E), corresponding to an approximate 30% reduction in mRNA levels compared with scrambled controls. The reduction correlated with a significant decrease in IFN‐γ production compared with scrambled controls (Figure 2F,G). ELISA assay for IFN‐γ production was chosen to provide a bulk quantitative measurement of total cytokine output following the gene‐silencing procedures. Together, pharmacological inhibition and gene silencing demonstrate that activation of TCRVβ2^+^ T cells depends on APC‐driven GSL biosynthesis, suggesting that endogenous GSLs act as functional lipid ligands.

**FIGURE 2:**
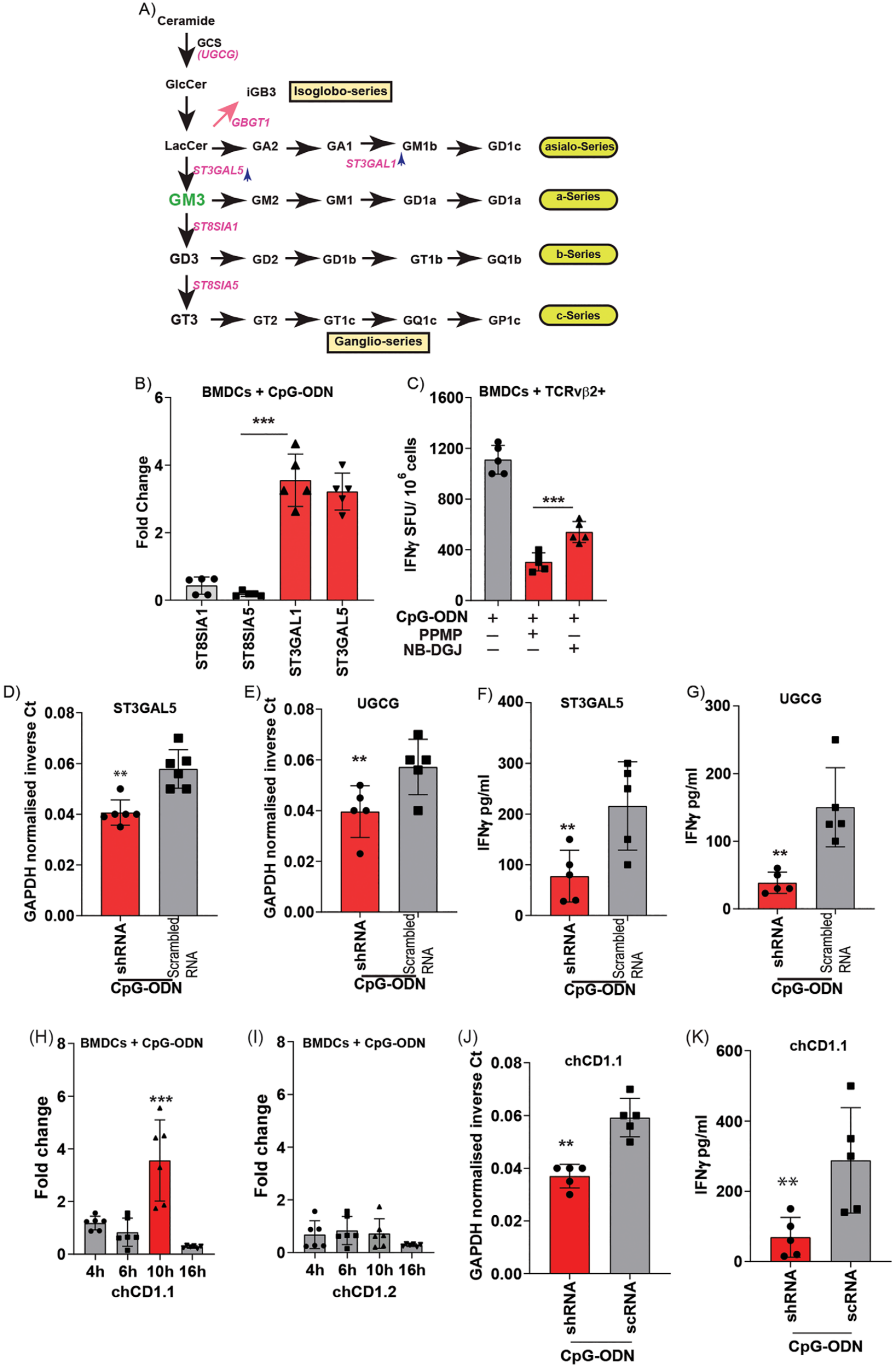
The UGCG/ST3GAL5/chCD1.1 axis regulates CpG‐ODN‐mediated T cell activation. This figure demonstrates that the activation of chicken TCRVβ2 T cells by CpG‐ODN‐stimulated APCs is dependent on the UGCG/ST3GAL5 biosynthetic pathway and the molecule chCD1.1. (A) A schematic outlines the biosynthetic pathway for glycosphingolipids (GSLs) from ceramide, with relevant enzymes indicated in italics. (B) mRNA expression levels of GSL‐associated enzymes in BMDCs 6 h post‐CpG‐ODN stimulation (1 µg/mL) show significant upregulation of *ST3GAL5* and *ST3GAL1* via qRT‐PCR, with levels shown as fold change relative to unstimulated controls. (C) Functional relevance is shown by the pretreatment of BMDCs with GSL synthesis inhibitors PPMP (10 µM) or NB‐DGJ (10 µM), which significantly reduces their ability to activate IFN‐γ production in TCRVβ2 T cells as measured by ELISpot. (D–E) Validation of shRNA‐mediated knockdown of **(D)** *ST3GAL5* and (E) *UGCG* in splenocytes is presented as GAPDH‐normalized inverse values, using scrambled RNA (scRNA) as a control. (F, G) Correspondingly, knockdown of (F) *ST3GAL5* and (G) *UGCG* significantly impairs CpG‐ODN‐induced IFN‐γ secretion in co‐culture with T cells. (H, I) A time‐course expression analysis in BMDC reveals that following CpG‐ODN treatment, significant upregulation is observed for (H) *chCD1.1* mRNA, peaking at 10 hours, while (I) *chCD1.2* remains unaffected. (J) Validation of shRNA‐mediated knockdown of *chCD1.1* in splenocytes is shown relative to scRNA controls. (K) Finally, knockdown of *chCD1.1* significantly impairs CpG‐ODN‐induced IFN‐γ secretion from splenocytes. For panels D, E, and J, the Y‐axis represents relative expression calculated as normalized to *GAPDH*. A reduction of approximately 30% in mRNA levels was observed for each target gene. Throughout the figure, data points represent individual chickens (at least *n* = 5), with bars representing the mean SD. Asterisks denote significant differences (**p < 0.01 and ***p < 0.001) as determined by one‐way ANOVA.

### 3.3 CD1.1 Is Involved in Presentation of Lipid Ligands to CD3^+^TCRVβ2^+^ T Cells

To investigate the role of chicken CD1 molecules (CD1.1 and CD1.2) in CpG‐ODN‐mediated activation of CD3^+^TCRVβ2^+^ T cells, we first assessed their expression in BMDCs by qRT‐PCR. CpG‐ODN stimulation significantly upregulated CD1.1 mRNA (∼fourfold, *p* < 0.001) after 10 h (Figure 2H), whereas CD1.2 expression remained unchanged (Figure 2I). CD1.1‐targeting shRNA significantly reduced CD1.1 mRNA expression by approximately 30% in electroporated splenocytes compared with scrambled controls (*p* < 0.01) (Figure 2J). Importantly, CD1.1 knockdown significantly reduced CpG‐ODN‐induced IFN‐γ production compared with controls (Figure 2K). Together, these results indicate that CD1.1 is required for optimal activation of CD3^+^TCRVβ2^+^ T cells in response to CpG‐ODN‐treated APCs, supporting its role in lipid antigen presentation. However, the identity of the activating ligands and potential co‐stimulatory requirements remain to be determined.

### 3.4 CD3^+^TCRVβ2^+^ T Cells Recognize Ganglioside GM3 and Produce NKT‐associated Cytokines

In mammals, endogenous glycosphingolipids (GSLs) generated in CpG‐ODN‐stimulated APCs can act as natural ligands for NKT cells [13]. To test whether similar ligands function in chickens, we examined whether GM3 or GM1 gangliosides, products of St3gal5 and St3gal1 activity, can activate chicken splenocytes. ELISpot analysis showed that GM3, but not GM1, induced IFN‐γ production (Figure 3A). We next assessed whether GM3‐pulsed BMDCs could activate specific T cell subsets. BMDCs loaded with GM3 were co‐cultured with purified CD3^+^, TCRVβ1^+^, or TCRVβ2^+^ T cells. IFN‐γ production was observed only in CD3^+^TCRVβ2^+^ T cells, indicating selective recognition of GM3‐presenting BMDCs (Figure 3B). To characterize the broader response, splenocytes stimulated with GM3 or CpG‐ODN were analyzed for cytokine and cytotoxic gene expression. GM3 significantly upregulated IL‐2, IL‐4, IL‐17, granzyme A (GZMA), and perforin A1 (PFA1) compared with controls (*p* < 0.05–0.01), with a stronger overall induction than CpG‐ODN (Figure 3C–G). Together, these data demonstrate that GM3 is an endogenous lipid ligand that activates CD3^+^TCRVβ2^+^ chicken T cells and induces a cytokine profile consistent with NKT‐like function in chickens. To confirm IFN‐γ production by TCRVβ2+ T cells following GM3 stimulation, we performed intracellular IFN‐γ staining on both TCRVβ1 and TCRVβ2 cells. The results demonstrated that TCRVβ2 T cells, but not TCRVβ1+ T cells, produced IFN‐γ following GM3 (20 µM) stimulation for 4 h (Figure 3H). A similar pattern was observed in two additional independent splenocyte preparations, with GM3 inducing IFN‐γ exclusively in TCRVβ2+ T cells. The increase in IFN‐γ‐producing TCRVβ2+ cells was 1% (net increase; 1.4%–0.4% = 1%) in the representative bird shown in Figure 3H, and 0.2% and 0.25% (net increase) in the two additional birds, respectively. While flow cytometry serves as qualitative confirmation of the cellular source of IFN‐γ, quantitative proof relies on their fully powered ELISpot and ELISA assays. A sample size of (*n* = 3) for IC assay limits statistical power due to individual variation. We believe that visualizing these individual data points alongside our robust ELISPOT data provides a transparent and faithful representation of the GM3‐specific response.

**FIGURE 3:**
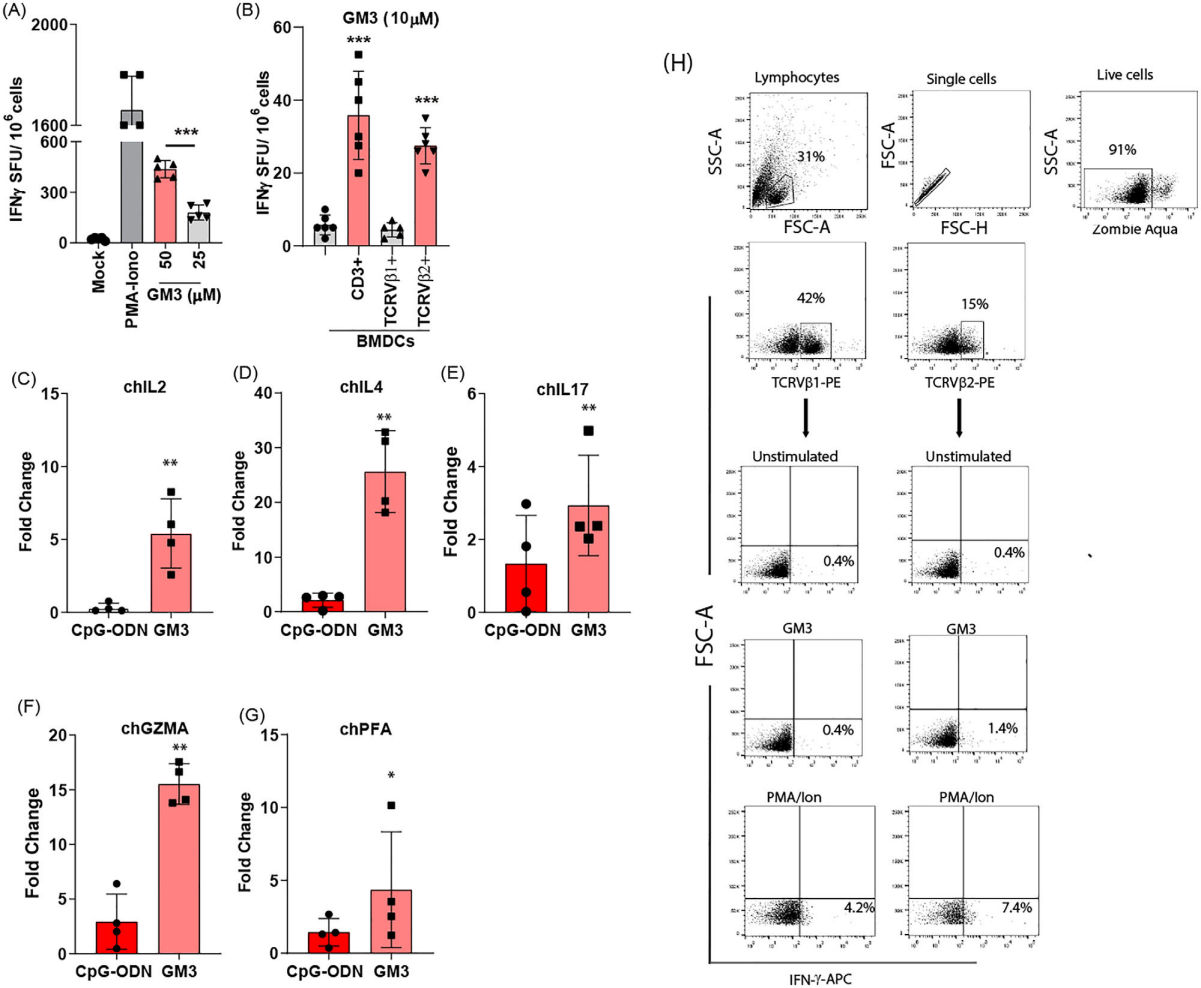
Chicken TCRVβ2 T cells recognize Ganglioside GM3 and exhibit a characteristic NKT‐like activation profile. (A, B) The frequency of IFN‐γ‐secreting cells was determined 24 h poststimulation with GM3 using an ELISpot assay. (A) Total chicken splenocytes were stimulated with exogenous Ganglioside GM3 (25 and 50 µM), PMA/Ionomycin as a positive control, or diluent as a negative control. (B) Purified CD3+, TCRVβ1+ or TCRVβ2+ T cells were co‐cultured with BMDCs pulsed with 10 µM GM3. (C–G) CpG‐ODN or GM3 stimulation induces a multifunctional cytokine and cytotoxic response in splenocytes. mRNA expression levels of (C) IL‐2, (D) IL‐4, (E) IL‐17, (F) Granzyme A (GZMA), and (G) Perforin (PRF1/PFA) were significantly elevated following GM3 treatment compared with CpG‐ODN stimulation. mRNA levels are presented as fold change relative to unstimulated controls after normalization to GAPDH. Each data point represents an individual chicken, and the graph represents the mean ± standard deviation of five independent replicates from each individual chicken; bars represent mean SD. Asterisks denote significant differences (*p < 0.05; **p < 0.01; and ***p < 0.001) determined by one‐way ANOVA. (H) Representative flow cytometry dot plots illustrating the gating strategy for splenocytes. The strategy includes sequential gating on lymphocytes, single cells, and live cells (identified by Zombie Aqua exclusion). Representative plots show the identification of TCRVβ1+ and TCRVβ2+ T cell subsets and their subsequent intracellular IFN‐γ production in unstimulated, PMA/Ion or GM3‐stimulated conditions. Numbers within the plots represent the percentage of cells within the designated gates

### 3.5 Recombinant IL‐12 and IL‐18 Induce IFN‐γ Production by CD3^+^TCRVβ2^+^ T Cells in the Presence of APCs

CpG‐ODN‐stimulated BMDCs showed significant upregulation of chIFN‐α, chIFN‐β, chIL‐12, and chIL‐18 by qRT‐PCR (Figure 4A). In ELISpot assays, only recombinant chIL‐12 and chIL‐18 induced IFN‐γ production in splenocytes, whereas chIFN‐α and chIFN‐β had minimal effects (Figure 4B). Purified TCRVβ1^+^ and TCRVβ2^+^ T cells did not respond to the recombinant cytokines alone. In co‐culture experiments, chIL‐12 and chIL‐18 selectively induced IFN‐γ production in CD3^+^TCRVβ2^+^ T cells, but not TCRVβ1^+^ cells, and this required live BMDCs. BMDCs in the absence of chIL‐12/IL‐18 did not induce IFN‐γ production in CD3^+^TCRVβ2^+^ T cells. Fixed APCs in the presence of chIL‐12/IL‐18 failed to support this response. Together, these data show that chicken IL‐12 and IL‐18 activate CD3^+^TCRVβ2^+^ T cells in an APC‐dependent manner (Figure 4C).

**FIGURE 4:**
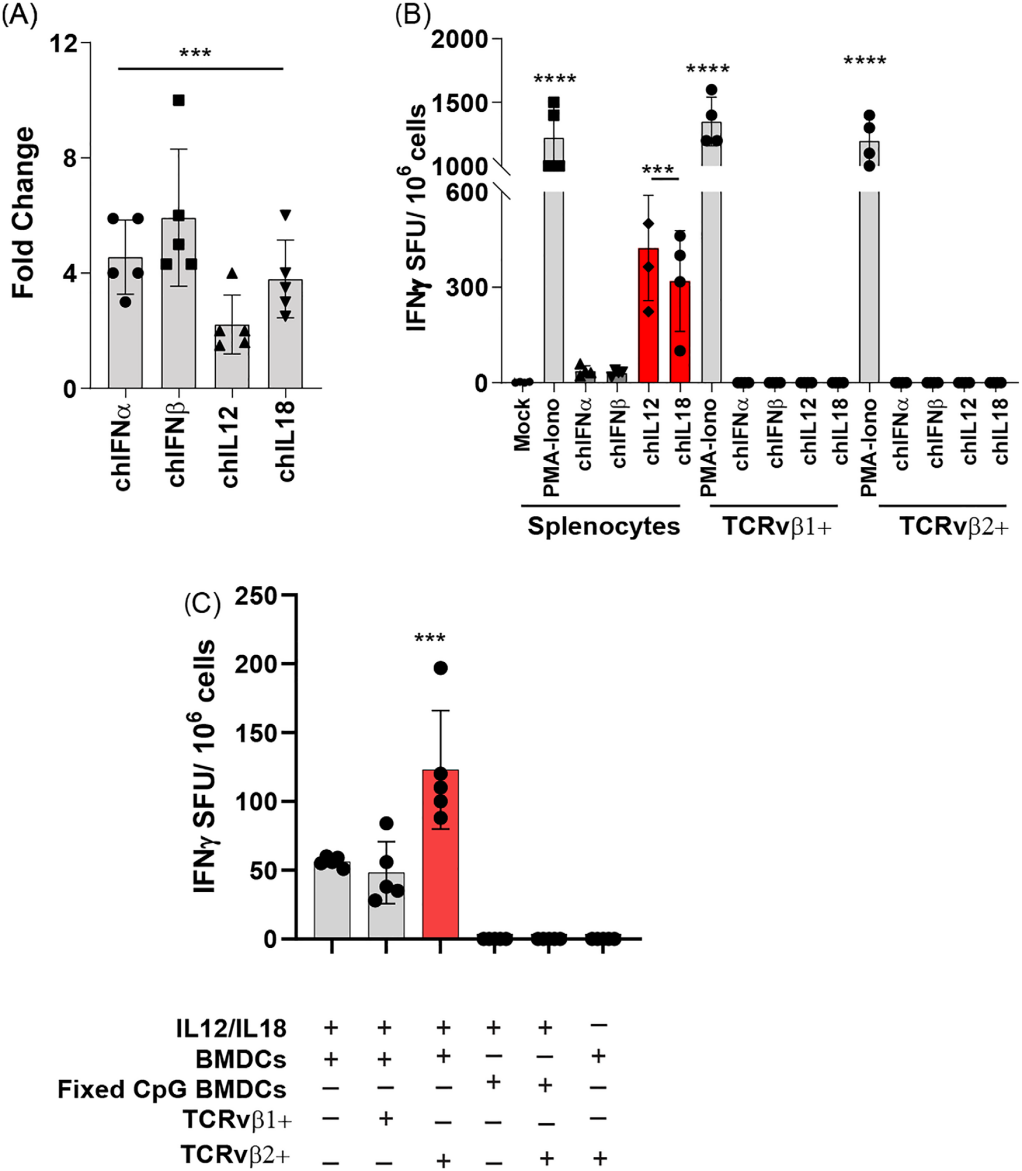
IL‐12 and IL‐18 stimulate IFN‐γ production from TCRVβ2+ T cells in the presence of live BMDCs. (A) Cytokine induction by CpG‐ODN. CpG‐ODN (1 µg/mL) treatment of BMDCs significantly upregulates the mRNA expression of chIL12, chIL18, chIFNα, and chIFNβ at 6 h post stimulation. mRNA levels are expressed as fold change relative to unstimulated BMDCs after normalization to GAPDH. (B) Direct stimulation assay. Recombinant chicken cytokines (IL‐12 or IL‐18) induced IFN‐γ production from splenocytes but failed to stimulate IFN‐γ production from purified chicken TCRVβ1or TCRVβ2 T cells. Cells were stimulated with diluent (mock control), PMA/Ionomycin (positive control), recombinant chicken IL‐12 (100 ng/mL), IL‐18 (100 ng/mL), IFN‐α (2000 IU/mL), or IFN‐β (2000 IU/mL) for 24 h, and the frequencies of IFN‐γ‐producing cells were determined using an ELISPOT assay. (C) IL‐12 and IL‐18 stimulate IFN‐γ production by TCRVβ2+, but not TCRVβ1+, T cells in the presence of live, unstimulated BMDCs. Unstimulated BMDCs were co‐cultured with TCRVβ1+ or TCRVβ2+ T cells in the presence of recombinant chicken IL‐12 (100 ng/mL) and IL‐18 (100 ng/mL) for 24 h. In some experiments, BMDCs were formaldehyde‐fixed before co‐culture with TCRVβ2+ T cells. Frequencies of IFN‐γ‐producing cells were determined using an ELISpot assay. Live BMDCs were required for IL‐12/IL‐18‐induced IFN‐γ production by TCRVβ2+ T cells. Data are a representation of three independent experiments; each performed with three biological replicates per treatment. Each dot represents the data from an individual chicken, and the graph represents the mean ± standard deviation of five independent replicates from individual chickens. Asterisks denotes significant differences (**p < 0.01; ***p < 0.001, and ****p < 0.0001) by ANOVA test.

## 4 Discussion

The data presented in this study provide the first functional evidence of a specialized population of innate‐like T cells in chickens that mirror the characteristics of mammalian invariant Natural Killer T (iNKT) cells. By demonstrating that CpG‐ODN‐activated antigen‐presenting cells (APCs) stimulate chicken TCRVβ2+ T cells through both lipid‐dependent and cytokine‐mediated pathways, we establish a conserved role for these cells in the avian immune system. This discovery highlights a significant functional divergence between chicken T cell subsets, which may be rooted in their distinct developmental origins. Specifically, TCRVβ1^+^ and TCRVβ2^+^ T cells are generated in the thymus as separate sublineages, with the development of the TCRVβ2^+^ lineage beginning approximately 3 days later than that of the TCRVβ1^+^ lineage [14]. It has been reported that the Vβ1 family contains a higher number of segments and encodes a consensus sequence corresponding to the mammalian Vβ subgroup I [15]. While we have previously shown that TCRVβ1+ T cells behave as conventional T cells, recognizing specific peptide epitopes within the pp38 antigen of Marek's disease virus (MDV) in an MHC‐restricted manner in both B19 (line P) and B21 haplotypes (line N) chickens [16], the current data suggest that the TCRVβ2+ subset is specialized for the recognition of nonpeptide lipid antigens. However, further research is required to establish whether this clear distinction between peptide‐reactive TCRVβ1 and nonpeptide‐reactive (e.g., lipids) TCRVβ2 populations represents a universal paradigm of the avian T cell repertoire.

A central finding of this research is that chicken T cell activation by CpG‐ODN requires the metabolic processing of endogenous lipids. In mammals, TLR9 stimulation triggers the generation of self‐glycosphingolipids (GSLs) that serve as TCR ligands [13, 17]. Our results show a parallel mechanism in chickens: CpG‐ODN upregulates the key biosynthetic enzymes *UGCG* and *ST3GAL5* (GM3 synthase) in BMDCs. The functional necessity of this pathway was confirmed by the fact that both chemical inhibition of GSL synthesis and shRNA‐mediated knockdown of *ST3GAL5* significantly impaired the ability of APCs to activate TCRVβ2+ T cells. It should be noted that the presented knockdown experiments do not distinguish APC‐intrinsic effects from broader effects on other splenocyte populations. Furthermore, we identified Ganglioside GM3 as a potent exogenous ligand and showed that GM3‐pulsed BMDCs specifically activate the TCRVβ2+ subset. This specificity suggests that the TCRVβ2 receptor in chickens may be functionally analogous to the semi‐invariant TCR used by mammalian iNKT cells to recognize CD1d‐presented lipids. The modest magnitude of IFN‐γ‐producing cells in the purified TCRVβ2^+^ T‐cell co‐culture relative to bulk splenocytes suggests that other splenic populations, such as NK cells or γδ T cells, may also be activated by GM3 or play a synergistic role in the total splenocyte response.

While chickens possess two CD1 isoforms (chCD1.1 and chCD1.2), our data suggest that chCD1.1 contributes to the activation of these NKT‐like cells. CpG‐ODN stimulation selectively induced *chCD1.1* mRNA, and its knockdown resulted in a significant reduction in T cell IFN‐γ production. However, the partial nature of this reduction and the observed variability suggest that CD1.1 may not be the sole dependency of this response, and other pathways could potentially contribute. We also recognize technical limitations in our assay for the validation of chCD1.1 at the protein level. While upregulation of mRNA was observed, we were unable to confirm upregulation of surface protein expression on CpG‐ODN‐activated BMDCs or knockdown using flow cytometry. Furthermore, functional blocking assays using CB3 antibody did not reduce T cell responses (data not shown). This lack of inhibition may be due to the CB3 epitope being located outside the TCR‐interaction site or the ligand‐binding groove, rather than an absence of CD1.1 involvement. A key limitation of our study is that direct involvement of the TCRVβ2 receptor in binding the CD1.1‐GM3 complex, or the clonal restriction of this cell population, was not demonstrated. Future studies utilizing TCR‐ or CD1.1‐blocking antibodies, TCRVβ2‐expressing reporter lines, or CD1.1‐expressing transfected cell lines are required to definitely establish the TCR role or CD1.1‐mediated restriction in avian lipid recognition.

This mimics the mammalian reliance on CD1d for iNKT activation and suggests that the structural features of chCD1.1 are evolutionarily optimized for the presentation of gangliosides like GM3. Consistent with mammalian models, chicken NKT‐like cells appear to be regulated by a “two‐signal” requirement. While the CD1.1‐lipid complex provides the primary TCR signal, CpG‐ODN also induces the secretion of IL‐12 and IL‐18 from APCs. We demonstrated that while these cytokines cannot stimulate purified T cells directly, they can drive robust IFN‐γ production in the presence of live APCs. Although macrophages have been shown to produce IFN‐γ [18], we are unable to rule out the possibility that IFN‐γ detected in BMM or BMDC cultures, in the absence of T cells, may originate, at least in part, from surviving chicken innate‐like lymphocytes such as NK cells or γδT cells following differentiation from bone marrow cells [19]. Interestingly, the failure of fixed APCs to support cytokine‐driven activation, even when exogenous IL‐12 and IL‐18 were provided, suggests that a membrane‐bound component or a baseline level of lipid presentation is still required for the cells to become responsive to cytokine signalling. While these observations point toward a strict requirement for direct cell‐to‐cell contact, such physical interaction was not explicitly demonstrated in this study. Definitive confirmation of this cell‐to‐cell contact mechanism remains a critical objective for future research. GM3‐stimulated splenocytes not only produced IFN‐γ but also expressed *chIL2*, *chIL4*, and *chIL17*, alongside cytotoxic markers like *Granzyme A* and *Perforin*. This multifunctional capacity allows them to potentially bridge the innate and adaptive branches of the avian immune system, much like their mammalian counterparts.

Targeting chicken TRVβ2+ NKT‐like cells through the incorporation of endogenous gangliosides (such as GM3) or synthetic glycosphingolipids (GSL) analogues as vaccine adjuvants represents a highly promising, classical MHC‐independent strategy to rapidly engage the avian innate‐adaptive immune bridge. Because CD1.1 is highly conserved and nonpolymorphic compared with classical chicken MHC molecules [20, 21], lipid adjuvanted vaccine formulations could provide a universal, host‐genotype‐independent platform to elicit protective immunity across genetically diverse, outbred populations. Based on mammalian models of NKT cell activation, mucosal delivery of GSLs could potentially trigger an immediate, localized cytokine response, including production of IFN‐γ and IL‐17 [1, 2, 3, 4, 5], cytokines we demonstrated to be expressed upon GM3 stimulation. These cytokines induce recruitment and activation of innate immune cells such as phagocytes, leading to an accelerated effector response, critical for clearing pathogens including *Salmonella* and *E. Coli* [22, 23]. The use of these vaccines could potentially reduce the poultry sector's reliance on prophylactic antibiotics and improve production efficiency, food security, and overall flock welfare.

In conclusion, this study identifies a TCRVβ2+ T cell population that recognizes endogenous and exogenous gangliosides in an APC‐dependent manner. These findings expand our understanding of avian immunology and provide a potential target for the development of lipid‐based adjuvants in poultry vaccines to enhance protection against viral and bacterial pathogens.

## Author Contributions

All authors contributed to drafting the manuscript or revising it critically for important intellectual content and approved the final version for publication. Nitin Kamble, Faisal Anjum, and Shahriar Behboudi were responsible for data acquisition, analysis, and interpretation, as well as study conception and manuscript preparation.

## Funding

This work was supported by research grants BB/T020954/1, BB/S01506X/1, and BBS/E/I/00007030 from the Biotechnology and Biological Sciences Research Council in the United Kingdom.

## Ethics Statement

No studies were performed on human samples. All experimental animal procedures were conducted according to Ethical approval from the Pirbright Institute and the Home Office project license (project license number P6257C392).

## Conflicts of Interest

The authors declare no conflicts of interest.

## Data Availability

The datasets generated and/or analyzed during the current study are available from the corresponding author upon reasonable request.
